# On the reliability of deep learning-based classification for Alzheimer’s disease: Multi-cohorts, multi-vendors, multi-protocols, and head-to-head validation

**DOI:** 10.3389/fnins.2022.851871

**Published:** 2022-09-07

**Authors:** Yeong-Hun Song, Jun-Young Yi, Young Noh, Hyemin Jang, Sang Won Seo, Duk L. Na, Joon-Kyung Seong

**Affiliations:** ^1^Department of Artificial Intelligence, Korea University, Seoul, South Korea; ^2^Department of Neurology, Gil Medical Center, Gachon University College of Medicine, Incheon, South Korea; ^3^Department of Neurology, Samsung Medical Center, School of Medicine, Sungkyunkwan University, Seoul, South Korea; ^4^School of Biomedical Engineering, Korea University, Seoul, South Korea; ^5^Interdisciplinary Program in Precision Public Health, College of Health Science, Korea University, Seoul, South Korea

**Keywords:** Alzheimer’s disease, multi-cohort validation, structural magnetic resonance imaging, deep learning, reliability, clinical application, low-resolution magnetic resonance imaging

## Abstract

Structural changes in the brain due to Alzheimer’s disease dementia (ADD) can be observed through brain T1-weighted magnetic resonance imaging (MRI) images. Many ADD diagnostic studies using brain MRI images have been conducted with machine-learning and deep-learning models. Although reliability is a key in clinical application and applicability of low-resolution MRI (LRMRI) is a key to broad clinical application, both are not sufficiently studied in the deep-learning area. In this study, we developed a 2-dimensional convolutional neural network-based classification model by adopting several methods, such as using instance normalization layer, Mixup, and sharpness aware minimization. To train the model, MRI images from 2,765 cognitively normal individuals and 1,192 patients with ADD from the Samsung medical center cohort were exploited. To assess the reliability of our classification model, we designed external validation in multiple scenarios: (1) multi-cohort validation using four additional cohort datasets including more than 30 different centers in multiple countries, (2) multi-vendor validation using three different MRI vendor subgroups, (3) LRMRI image validation, and finally, (4) head-to-head validation using ten pairs of MRI images from ten individual subjects scanned in two different centers. For multi-cohort validation, we used the MRI images from 739 subjects from the Alzheimer’s Disease Neuroimaging Initiative cohort, 125 subjects from the Dementia Platform of Korea cohort, 234 subjects from the Premier cohort, and 139 subjects from the Gachon University Gil Medical Center. We further assessed classification performance across different vendors and protocols for each dataset. We achieved a mean AUC and classification accuracy of 0.9868 and 0.9482 in 5-fold cross-validation. In external validation, we obtained a comparable AUC of 0.9396 and classification accuracy of 0.8757 to other cross-validation studies in the ADNI cohorts. Furthermore, we observed the possibility of broad clinical application through LRMRI image validation by achieving a mean AUC and classification accuracy of 0.9404 and 0.8765 at cross-validation and AUC and classification accuracy of 0.8749 and 0.8281 at the ADNI cohort external validation.

## Introduction

Alzheimer’s disease dementia (ADD) is a degenerative neurological disease that causes disability in the overall performance of daily life due to the gradual decline of neurological function. Since neurodegeneration occurs before the decline of neurological function, brain T1-weighted magnetic resonance imaging (MRI) was used for diagnosing ADD. In this context, many researchers studied MRI-based computer-aided ADD diagnostic with machine learning (Magnin et al., 2009; Cho et al., 2012; Gray et al., 2013). Within the success of convolutional neural networks (CNNs), several ADD diagnostic studies have been conducted (Aderghal et al., 2017; Liu et al., 2018; Bae et al., 2020; Yee et al., 2021; Zhang et al., 2021) with CNN in recent years due to several advantages: CNN can learn meaningful features from data without a feature extraction preprocessing process. In addition, transfer learning can be employed to develop a model with high performance when the amount of data used for training is limited.

Reliability is essential when transferring technology to clinical applications. However, the heterogeneity of MRI scan parameter settings (e.g., repeat time, echo time, and slice thickness), MRI scanner vendors, and several factors cause the diverse-protocol problem. This problem causes bias in the MRI-derived features and the prediction of CNN models (Han et al., 2006; Schnack et al., 2010; Mårtensson et al., 2020). Several methods have been suggested for the MRI-derived features to solve the issue by harmonizing features (Chung et al., 2017; Fortin et al., 2018; Ma et al., 2019). However, the diverse-protocol problem has not yet been studied sufficiently in the deep learning-based classification of brain disease.

Moreover, the type of clinically applicable MRI protocols is also important. Most deep learning studies focus on 3-dimensional (3D) T1-weighted MRI, which we referred to as high-resolution MRI (HRMRI). HRMRI takes more time and costs than 2-dimensional (2D) T1-weighted MRI, which we referred to as low-resolution MRI (LRMRI). LRMRI images are scanned more frequently in the clinic than HRMRI images due to these advantages. Thus, the reliability of LRMRI image-based prediction should be considered from a broad clinical application perspective.

In this study, we sought to find surrogate solutions for the reliability problem by developing robust deep learning methods with comprehensive validation using multiple possible scenarios. A new 2D CNN-based deep learning model was developed based on the pretrained ImageNet weights (Deng et al., 2009) with label smoothing (Müller et al., 2019). Our model further adopted several methods for reliability across heterogeneous MRI scan types. Candidate methods are as follows: individual slice encoding to extract sufficient information from each slice and to lessen the effect of slice thickness, which is noticeable in a three-dimensional setting. On the assumption that providing multiple slices at once lets CNN models learn the relationship between the slices and causes degradation of robustness in various slice thickness settings, we encoded each slice individually and concatenated feature vectors before the fully connected layer, rather than concatenating several slices in the channel axis. Instance normalization performs contrast normalization on its input (Ulyanov et al., 2016). It has been known that instance normalization performs style normalization (Huang and Belongie, 2017), which helps learn features invariant to the style (Pan et al., 2018). Mixup augments data by convex combination of samples and their labels, which improves model performance and helps train a model with data that have noise on its label (Zhang et al., 2017). Sharpness-aware minimization (SAM) finds flat minima by adding regularization terms related to sharpness (Foret et al., 2020). By searching flat minima, SAM can find weights that generalize well than the usual approach.

To validate the efficacy of the proposed methods, multiple scenarios were designed, including multi-cohort validation, multi-vendor validation, and head-to-head validation. Furthermore, for broad clinical application, we generated LRMRI images from HRMRI images in each cohort and further performed LRMRI image validation for every validation scenario.

## Materials and methods

### Participants and magnetic resonance imaging acquisitions

We used the MRI images from 5,204 subjects from six different cohorts in this study to train and extensively validate and evaluate model development.

#### Participants

The Samsung medical center (SMC) cohort consisted of 2,765 cognitive normal (CN) subjects and 1,192 ADD subjects who have undergone the 3D T1-weighted turbo field echo MRI scans using the Philips 3T MRI scanner. The Alzheimer’s Disease Neuroimaging Initiative (ADNI) cohort consisted of 453 CN subjects and 286 ADD subjects. Each subject in the ADNI cohort had undergone 3D T1-weighted MRI scans using one of the six protocols. The Dementia Platform Korea project (DPKR) cohort consisted of 67 CN subjects and 58 ADD subjects. Each subject in the DPKR cohort had undergone 3D MRI scans using one of the four protocols. The Premier (PRM) cohort consisted of 69 CN subjects and 165 ADD subjects who have undergone 3D T1-weighted MRI scans using the same protocol as the SMC cohort case. The Gachon medical center (GMC) cohort consisted of 61 CN and 78 ADD subjects who have undergone 3D T1-weighted magnetization-prepared rapid gradient-echo MRI scans using the Siemens 3T scanner. The SMC/Chaum cohort consisted of 10 subjects who had undergone two 3D T1-weighted MRI scans, one using the same protocol in the SMC cohort case and the other using the Chaum’s.

The MRI images belonged to one vendor subgroup: GE, Philips, and Siemens. A total of 352, 353, and 307 MRI images from subjects belong to the GE, Philips, and Siemens vendor subgroups, respectively. We excluded MRI images from subjects from the SMC and SMC/Chaum cohorts in the vendor subgroups. Except for the SMC/Chaum cohort, we used only one MRI image per subject. Table 1 shows the detailed information of subjects per diagnostics group, MRI scan protocol, and MRI scanner vendors.

**TABLE 1:** The number of subjects used in this study.

Cohort		Vendor	Tesla	Imaging technique	Slice thickness (mm)	Repeat time (ms)	Echo time (ms)	Flip angle (°)	*N*_*CN*_	*N*_*ADD*_	*N*_*total*_
SMC		Philips	3.0	TFE	0.5	9.9	4.6	8	2765	1192	3957
Alzheimer’s disease neuroimaging initiative (ADNI)		GE	1.5	MPRAGE	1.2	8.6–10.4	3.8–4.1	8	105	88	193
			3.0	IR-FSPGR	1.2	7–7.68	2.8–3.2	11	91	47	138
		Philips	1.5	MPRAGE	1.2	8.6	4	8	8	8	16
			3.0	MPRAGE	1.2	6.7	3.1	9	13	5	18
		Siemens	1.5	MPRAGE	1.2	2400–3000	3.5–3.9	8	91	68	159
			3.0	MRPAGE	1.2	2300	3.0	9	145	70	215
		
		Total							453	286	739

Dementia platform Korea project (DPKR)		GE	3.0	IR-FSPGR	1.0–1.3	7.4–8.2	2.7–3.2	11–12	13	8	21
		Philips	3.0	TFE	0.5–1.2	6.7–9.4	3.1–4.6	8–9	40	40	80
			3.0	MPRAGE	1.0	8.3	3.8	8	4	1	5
		Siemens	3.0	MPRAGE	1.0–1.26	1470–2300	2.3–3.9	9–15	10	9	19
		
		Total							67	58	125

Premier (PRM)		Philips	3.0	TFE	0.5	9.9	4.6	8	69	165	234

Gachon medical center (GMC)		Siemens	3.0	MPRAGE	1.0	1900	2.9	8	61	78	139

SMC/Chaum	SMC	Philips	3.0	TFE	0.5	9.9	4.6	8	-	-	10
	Chaum	GE	1.5	Ax BRAVO	0.5	9.1	3.6	12	-	-	10
		
		Total							-	-	10

Total									3415	1779	5204

It is noted that the number of subjects whose MRI image registration was failed is not included in this table. MRI images from the SMC cohorts are used for a train set and LRMRI image validation. MRI images from the SMC/Chaum cohorts are used for head-to-head validation. MRI images from the other cohorts are used for multi-cohorts, multi-vendors, and LRMRI image validation.

MRI, magnetic resonance imaging; SMC, Samsung medical center; LRMRI, low-resolution MRI; N, number of subjects; TFE, turbo field echo; MPRAGE, magnetization-prepared rapid gradient-echo; IR-FSPGR, inversion recovery–prepared fast spoiled gradient-echo; Ax BRAVO, axial 3-dimensional brain volume; CN, cognitive normal; ADD, Alzheimer’s disease dementia.

The MRI images from the SMC cohort’s subjects were used for model training. MRI images from subjects belonging to the ADNI, DPKR, PRM, and GMC were used for external validation. Moreover, ten pairs of MRI images from subjects in the SMC/Chaum cohort were used for head-to-head validation.

#### Low-resolution magnetic resonance imaging image generation

**FIGURE 1:**
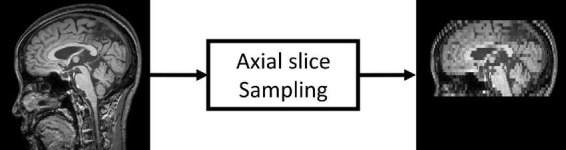
Low-resolution magnetic resonance imaging (LRMRI) image generation. LRMRI images were generated from high-resolution magnetic resonance imaging (HRMRI) images in this study by sampling a maximum total of 25 slices in an axial plane containing cerebral in uniform intervals. After sampling, the slice thickness becomes 0.5–1.25 mm to 4.5–5.5°mm.

Since most public MRI datasets consist of HRMRI images, we generated LRMRI images by downsampling axial slices of non-preprocessed original HRMRI images to perform LRMRI image validation. We sampled a maximum total of 25 slices in an axial plane containing cerebral from original HRMRI images with uniform intervals. Figure 1 shows the example of the input HRMRI image and the output LRMRI image. After downsampling, slice thickness becomes 0.5–1.25mm to 4.5–5.5mm, and the imaging plane was converted to an axial plane from the sagittal plane. We used the downsampled template and skull mask to preprocess downsampled images. After image preprocessing, we checked its quality and excluded 150 subjects due to registration failure. Table 2 shows the detailed information of the MRI images from subjects per diagnostics group, MRI scan protocol, and MRI scanner vendors.

**TABLE 2:** The number of subjects whose low-resolution magnetic resonance imaging image registration was succeeded.

Cohort	Vendor	Tesla	Imaging technique	Slice thickness (mm)	Repeat time (ms)	Echo time (ms)	Flip angle (°)	*N*_*CN*_	*N*_*ADD*_	*N*_*total*_
SMC	Philips	3.0	TFE	4.5	9.9	4.6	8	2765	1186	3951
Alzheimer’s disease neuroimaging initiative (ADNI)	GE	1.5	MPRAGE	4.7–5.5	8.6–10.4	3.8–4.1	8	104	88	192
		3.0	IR-FSPGR	5.1	7–7.68	2.8–3.2	11	91	47	138
	Philips	1.5	MPRAGE	4.7–4.9	8.6	4	8	8	8	16
		3.0	MPRAGE	5.0	6.7	3.1	9	13	5	18
	Siemens	1.5	MPRAGE	5.0–5.4	2400–3000	3.5–3.9	8	91	68	159
		3.0	MRPAGE	4.9–5.5	2300	3.0	9	145	70	215
	
	Total							452	286	738

Dementia platform Korea project (DPKR)	GE	3.0	IR-FSPGR	4.5–5.0	7.4–8.2	2.7–3.2	11–12	13	8	21
	Philips	3.0	TFE	4.5–5.0	6.7–9.4	3.1–4.6	8–9	25	24	49
		3.0	MPRAGE	5.0	8.3	3.8	8	1	4	5
	Siemens	3.0	MPRAGE	4.5–5.0	1470–2300	2.3–3.9	9–15	10	9	19
	
	Total							49	45	94

Premier (PRM)	Philips	3.0	TFE	4.5	9.9	4.6	8	2	1x26	128

Gachon medical center (GMC)	Siemens	3.0	MPRAGE	5.0	1900	2.9	8	61	78	139

Total								3329	1721	5050

It is noted that MRI images were generated from HRMRI images.

MRI, magnetic resonance imaging; SMC, Samsung medical center; LRMRI, low-resolution MRI; HRMRI, high-resolution MRI; N, number of subjects; TFE, turbo field echo; MPRAGE, magnetization-prepared rapid gradient-echo; IR-FSPGR, inversion recovery–prepared fast spoiled gradient-echo.

### Image preprocessing

**FIGURE 2:**
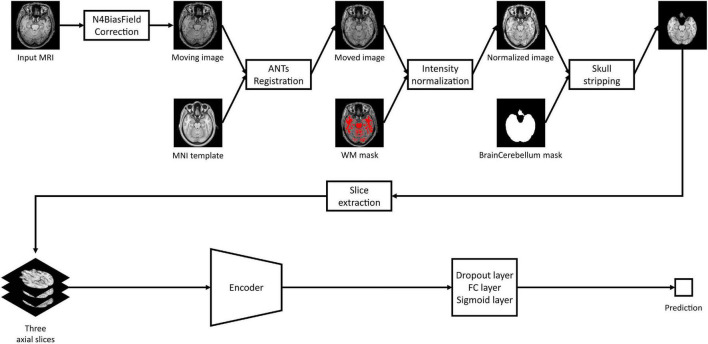
Study overview. Magnetic resonance imaging images are preprocessed with our preprocessing pipeline, and three axial slices including the hippocampus were extracted. The extracted slices are converted into scalar prediction by passing through the encoder, dropout, fully connected, and sigmoid layers.

As shown in Figure 2, all MRI images including both HRMRI and LRMRI were first non-uniformly corrected using the N4ITK algorithm (Tustison et al., 2010) and non-linearly registered to MNI space using the Advanced Normalization Tools (ANTs) software package (Avants et al., 2009). After registration, all MRI images were skull stripped, and their intensity has trimmed by the sum of the mean intensity of the white matter (WM) mask and 2.5 times the standard deviation of the WM mask, where the WM mask was calculated using the fuzzy C-means clustering algorithm (Reinhold et al., 2019). Finally, MRI images were min-max normalized, and then three axial slices, including the hippocampus region, were extracted to use ImageNet pretrained weights.

### Model development

We used the Inception Res Net V2 (Szegedy et al., 2017) architecture as a backbone since it is fast and lightweight and performs well. To access the effect of model developments, we trained five versions of the models, models A, B, C, D, and E, in which all of their encoders have the same input size of 130 × 130 × 3.

Model A is a vanilla Inception Res Net V2 model without a top. We added a dropout layer with a 0.2 dropout ratio, a fully connected layer with one output node, and a sigmoid activation layer at the end of model A. For model A, the three preprocessed slices were concatenated into a channel axis, encoded into a 1,536-dimensional vector, passed three layers, and created a scalar prediction which means the probability of ADD that can be used in classification when the threshold is applied. Model A structure would be a usual choice for researchers who plan to use pretrained weights. Model B is the same as model A except for MRI slice encoding. To encode enough information from each slice individually and combine them at a high level, we encoded each of the three axial slices from one subject and concatenate them before the fully connected layer. The three preprocessed slices were converted into a total of three individual three-channel images by duplicating its single-channel image three times. After converting, three images were encoded into three 1,536-dimensional vectors, concatenated into a 4,608-dimensional vector, and passed the three layers mentioned above. Model C is the same as model B except for its normalization layer. Instance normalization was used instead of batch normalization (Ioffe and Szegedy, 2015). Model D is the same as model C except for the training procedure. Mixup (Zhang et al., 2017) with its hyperparameter of 10 was used in model D. Model E is the same as model D except for the optimizing process. We used SAM when optimizing model E. As a result, A, B, and C each have their own structure and the same optimizing procedure, but C, D, and E all have the same structure and different optimizing procedures.

Also, ImageNet pretrained weights except for the normalization layers were adopted to speed up model training. We trained all models for 100 epochs with a batch size of 64, optimized through SGD optimizer with the momentum of 0.9, and label smoothing (Müller et al., 2019) with its hyperparameter of 0.1 using weighted binary cross-entropy loss. Left and right flip was performed for real-time data augmentation. For each of the five models, a 5-fold cross-validation was conducted with the SMC cohort. The SMC cohort’s MRI scans were randomly divided into five groups, four of which were used for training and the remaining for validation. For the random split, the random seed 0 was used. We saved weights with the best validation area under the receiver operating curve (AUC) at the end of an epoch for each fold. Then, we averaged the predictions of the models obtained by 5-fold cross-validation, from models A to E when evaluating the model with external cohorts.

All training was conducted using NVIDIA P100 GPUs with 16GB of memory per GPU, and all deep learning models were implemented using Tensorflow version 2.5.0 (Abadi et al., 2016) and Keras version 2.5.0 (Chollet, 2015).

### The design of multi-scenario validation

We developed the following hypotheses concerning an ideal trustworthy MRI-based ADD classification model: an ideal reliable MRI-based ADD classification model would not have poor performance when given unseen protocol MRI, would be robust to scanner vendor differences, would be robust to LRMRI images, and would have the same prediction values when given MRI scans from the same subject at similar time points. We built multi-scenario validation to analyze how our models satisfy those hypotheses in various conditions.

#### Multi-cohort, multi-vendor, and low-resolution magnetic resonance imaging image validation

We designed multi-cohort validation with the ADNI, DPKR, PRM, and GMC cohorts. MRI images from the PRM cohort have the same protocol as the SMC cohort, while the other cohorts have different protocols. Thus, we evaluated the model’s reliability by comparing the two model’s performance in the ADNI, DPKR, and GMC cohorts. Also, we would interpret the effect of applied methods on the model’s classification performance by comparing the model’s performance to the PRM cohort.

Similarly, we designed multi-vendor validation with the GE, Philips, and Siemens vendor subgroups. For multi-vendor validation, we would interpret the effect of applied methods on the model’s reliability by comparing the two model’s performance on the GE and Siemens vendor subgroups. Also, we would interpret the effect of applied methods on the model’s classification performance by comparing the model’s performance on the Philips vendor subgroup.

To evaluate the model’s performance on LRMRI images, we designed LRMRI image validation with the generated LRMRI images. We repeated multi-cohort validation and multi-vendor validation with LRMRI images. It is noted that the input image’s size is not changed from 130 × 130 × 3 when evaluating the models on LRMRI images.

Two metrics were used to assess performance: AUC and classification accuracy. The classification accuracy was determined using Youden’s J statistics (Youden, 1950), which determines the cutoff point for the maximum summation value of specificity and sensitivity.

#### Head-to-head validation

To explore the reproducibility of the model at the subject level, we performed head-to-head validation with the SMC/Chaum cohort. Subjects in the SMC/Chaum have paired MRI images: one is from the SMC and the other one is from the Chaum, and the mean time interval between the two scans was 300°days. We calculated two metrics, namely, Δ*prediction* and *slope*,


$$\Delta \,p\,r\,e\,d\,i\,c\,t\,i\,o\,n=\frac{1}{n}\,\sum_{i=1}^{n}\left|p\,r\,e\,d\,i\,c\,t\,i\,o\,n_{i}^{\text{SMC}}-p\,r\,e\,d\,i\,c\,t\,i\,o\,n_{i}^{\text{Chaum}}\right|$$

$$s\,l\,o\,p\,e=\min\left(\alpha ,\frac{1}{\alpha }\right),$$

where *prediction*^SMC^ = the model’s prediction of the images scanned from the SMC; *prediction*^Chaum^ = the model’s prediction of the images scanned from the Chaum; *i* = the index of the subject; and α = the value obtained from the slope of the linear regression equation of the model’s predictions. We could interpret a model with a smaller Δ*prediction* value, and a bigger *slope* value would be more reliable.

## Results

### Cross-validation

**FIGURE 3:**
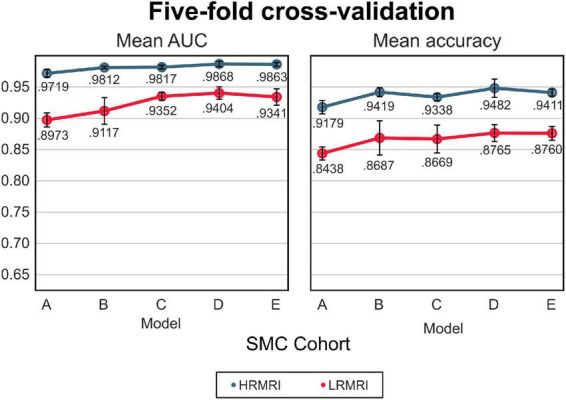
The 5-fold cross-validation and low-resolution magnetic resonance imaging image validation with the samsung medical center cohort. The blue line indicates the result of the high-resolution magnetic resonance imaging images, while the red line indicates the result of the LRMRI images. Models: A = vanilla inception res net V2; B = same with A except for slice encoding. Instead of encoding three slices at once, each of the three is encoded; C = same with B except for the normalization layer. Instead of batch normalization, the instance normalization layer was used; D = same with C except for the training procedure. Mixup with its hyperparameter value of 10 was used; E = same with D except for the optimizing process. Sharpness-aware minimization was used. SMC, Samsung medical center; HRMRI, high-resolution magnetic resonance imaging; LRMRI, low-resolution magnetic resonance imaging.

The mean AUC and the mean classification accuracy across 5-fold are plotted in Figure 3, the blue line. The performance gap was the biggest between model A and model B, respectively, 0.0093 and 0.024 for the mean AUC and classification accuracy. From model B to model E, the gap was relatively small.

### Exploring the effect of modification on reliability *via* multi-scenario validation

We assessed our five models with multi-scenario validation. Since the models are the same as adjacent versions of the models except for one option, we explored the effect of model modifications on reliability by comparing two adjacent versions of the models, e.g., model B and model C are the same but differ in their normalization layers. By comparing two adjacent models, for example, we can assess the effect of the instance normalization vs. the batch normalization.

#### Multi-cohort, multi-vendor, and low-resolution magnetic resonance imaging image validation

**FIGURE 4:**
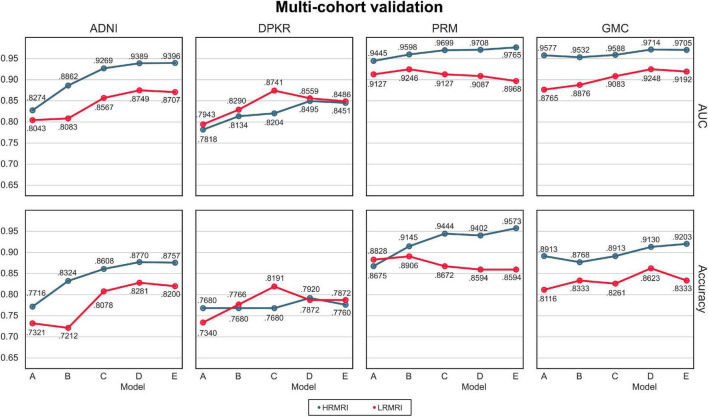
Multi-cohorts and low-resolution magnetic resonance imaging image validation. All the results are calculated by averaging the predictions of the models obtained from 5-fold cross-validation. The blue line indicates the result of the high-resolution magnetic resonance imaging images, while the red line indicates the result of the LRMRI images. Models: A = vanilla inception res net V2; B = same with A except for slice encoding. Instead of encoding three slices at once, each of the three is encoded; C = same with B except for the normalization layer. Instead of batch normalization, the instance normalization layer was used; D = same with C except for the training procedure. Mixup with its hyperparameter value of 10 was used; E = same with D except for the optimizing process. Sharpness-aware minimization was used. ADNI, Alzheimer’s Disease Neuroimaging Initiative; DPKR, Dementia Platform Korea project; PRM, Premier; GMC, Gachon medical center; HRMRI, high-resolution magnetic resonance imaging; LRMRI, low-resolution magnetic resonance imaging.

**FIGURE 5:**
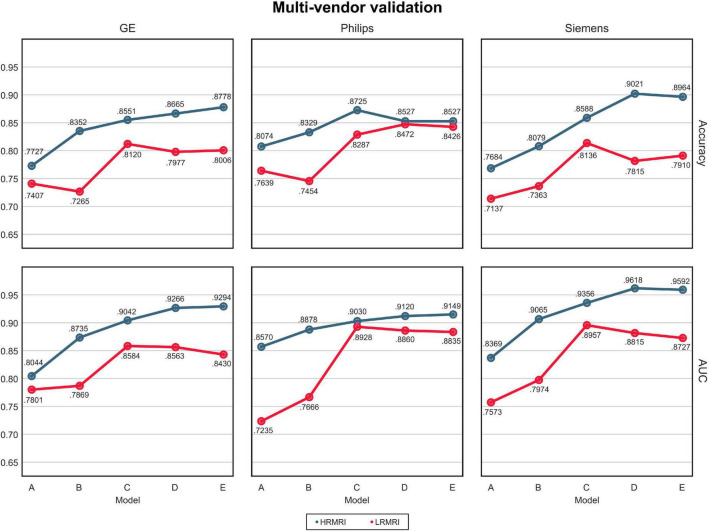
Multi-vendors and low-resolution magnetic resonance imaging image validation. The values in this figure were calculated by averaging the predictions of the models obtained from 5-fold cross-validation. The blue line indicates the result of the high-resolution magnetic resonance imaging, while the red line indicates the result of the LRMRI. Models: A = vanilla inception res net V2; B = same with A except for slice encoding. Instead of encoding three slices at once, each of the three is encoded; C = same with B except for the normalization layer. Instead of batch normalization, the instance normalization layer was used; D = same with C except for the training procedure. Mixup with its hyperparameter value of 10 was used; E = same with D except for the optimizing process. Sharpness-aware minimization was used. HRMRI, high-resolution magnetic resonance imaging; LRMRI, low-resolution magnetic resonance imaging.

The AUC and classification accuracy for four cohorts are plotted in Figure 4, the blue line. Moreover, the AUC and classification accuracy for three vendor subgroups is plotted in Figure 5, blue line. The overall AUC and classification accuracy increases from model A to model E for all cohorts and vendor subgroups, which means that the modifications enhanced the model’s classification performance. This implies that the modifications are not only successful in the perspective of the model’s classification performance but also successful in the perspective of the model’s reliability.

For the LRMRI image validation, the mean AUC and the mean classification accuracy across 5-fold are plotted in Figure 3, the red line. It is noted that we trained the models with only HRMRI images. In this study, it is hard to tell which models C, D, or E classify LRMRI better.

For multi-cohort validation, the results are plotted in Figure 4, the red line. In addition, for multi-vendor validation, the results are plotted in Figure 5, the red line. In this study, the overall tendency follows the above multi-cohort validations without the PRM cohort since the generated LRMRI images in the PRM cohort are poor-balanced: the PRM cohort contains only two LRMRI images for the CN subjects. The overall tendency in the multi-vendor validation also follows the multi-vendor validation performed above.

#### Head-to-head validation

**FIGURE 6:**
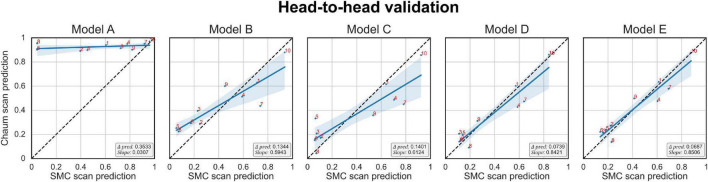
Head-to-head validation. The black dotted line indicates y = x. Subjects in the SMC/Chaum cohort have undergone two magnetic resonance imaging scans, one at the SMC and the other at the Chaum. The x-axis represents the averaged predictions of the SMC scans from the models obtained from 5-fold cross-validation, while the y-axis represents the averaged predictions of the Chaum scans from the models obtained from 5-fold cross-validation. Δ*pred* is the mean of the absolute value of the difference between the predictions for the SMC scans and the Chaum scans. The *slope* is the smaller of the two values, α and $\frac{\alpha }{1}$, where α is the value obtained from the slope of the linear regression equation of the predictions from the SMC scans and the Chaum scans. The blue line indicates the result of linear regression. The smaller Δ*pred* and the bigger *slope* are better. Models: A = vanilla inception res net V2; B = same with A except for slice encoding. Instead of encoding three slices at once, each of the three is encoded; C = same with B except for the normalization layer. Instead of batch normalization, the instance normalization layer was used; D = same with C except for the training procedure. Mixup with its hyperparameter value of 10 was used; E = same with D except for the optimizing process. Sharpness-aware minimization was used. SMC, Samsung medical center.

The Δ*prediction*, *slope*, and the prediction score of each subject per model are plotted in Figure 6. The *x*-axis is the model’s prediction score of the SMC scans, while the *y*-axis is for the Chaum scans. From models A to E, Δ*prediction* decreases, and *slope* increases, meaning that the modifications enhanced the model’s reliability.

## Discussion

In this study, we constructed an MRI-based ADD classification CNN model using individual slice encoding, instance normalization, Mixup, and SAM and showed that the model’s reliability could be improved by adopting those methods. We further validated the efficacy of the proposed model using comprehensive multi-scenarios: multi-protocols, multi-vendors, LRMRI image, and head-to-head validation that are critical in the usage of our tool in the real-world clinical field.

*Via* multi-scenario validation, we extensively investigated the effect of each option in terms of reliability across heterogeneous environments: (1) the individual slice encoding method improved the model’s performance in various experimental settings. The individual slice encoding method integrates information at a higher level, thereby the model can extract more reliable information related to ADD and avoid overfitting, and (2) the instance normalization method increased the model’s reliability by learning invariant features to different styles (Pan et al., 2018). In our problem setting, the features learned by different styles could be related to different MRI scan protocols. Therefore, the instance normalization technique makes our model robust against different MRI protocols, and (3) the Mixup method sometimes failed to improve the model’s performance on LRMRI if augmented data are different from test data. But it improved the model’s performance in most cases by generating a convex combination of the input samples, which would approximate various intermediate stages of disease progression, and (4) the SAM technique failed to improve the model’s performance on LRMRI when the sharp minima which skipped to find the flat minima performed better in test data than flat minima. However, it increased the model’s reliability in most cases by finding weights that are more robust to distributional shifts, which might be related to heterogeneous MRI scan protocols.

We compared the performance of the proposed method with other previous deep learning-based ones (Table 3). The data used for training or validation are different from each other, so it is not comfortable to directly compare the performance in detail. Nevertheless, we can see that our model E achieved comparable performance with other studies, even though our model E had not seen protocols in the ADNI cohort during the training process.

**TABLE 3:** Comparison of published cognitive normal vs. Alzheimer’s disease dementia classification performances in the Alzheimer’s disease neuroimaging initiative cohort.

Study		AUC	Acc	*N*_*CN*_	*N*_*ADD*_	*N*_*Total*_	Evaluation scheme	Description
Aderghal et al. (2017)		-	0.9141	228	188	416	Single split	2D CNN

Liu et al. (2018)		0.9586	0.9109	200	159	359	ADNI1 train, ADNI2 test	Landmark-based 3D CNN

Yee et al. (2021)		0.945	0.881	423	330	753	5-fold CV	3D CNN

Bae et al. (2020)		0.94	0.89	195	195	390	5-fold CV	2D CNN
		0.88	0.83				External validation	

Zhang et al. (2021)		0.984	0.913	231	200	431	5-fold CV	3D CNN

Ours, model	A	0.8274	0.7716	453	286	739	External validation	2D CNN
	B	0.8862	0.8324					
	C	0.9269	0.8608					
	D	0.9389	0.8770					
	E	0.9396	0.8757					

Models: A = vanilla inception res net V2; B = same with A except for slice encoding. Instead of encoding three slices at once, each of the three is encoded; C = same with B except for the normalization layer. Instead of batch normalization, the instance normalization layer was used; D = same with C except for the training procedure. Mixup with its hyperparameter value of 10 was used; E = same with D except for the optimizing process. Sharpness-aware minimization was used.

CN, cognitive normal; ADD, Alzheimer’s disease dementia; ADNI, Alzheimer’s Disease Neuroimaging Initiative; AUC, area under the receiver operating curve; Acc, accuracy; N, number of subjects used for the test; 2D, 2-dimensional; 3D, 3-dimensional; CNN, convolutional neural networks; CV, cross-validation.

Our LRMRI image validation has limitations since the generated LRMRI images were used as the surrogate of the original LRMRI images. Nevertheless, the performance could be meaningful because it used an image that reflects low resolution in its axial axis of LRMRI images, a significant difference between LRMRI image and HRMRI image.

In conclusion, we showed that we could improve a CNN model’s reliability with a small computational cost by adopting minor modifications to the existing deep learning model, which was then extensively validated through multiple scenarios. The proposed method would be worth applying to researchers who want to conduct CNN-based ADD diagnostic research in various experimental settings. Our study can potentially be improved further by designing additional real-world encounterable validation scenarios.

## Data availability statement

The datasets generated and/or analyzed for this study are available from the corresponding author, Prof. J-KS on reasonable request. Requests to access these datasets should be directed to jkseong@korea.ac.kr.

## Ethics statement

Ethical review and approval was not required for the study on human participants in accordance with the local legislation and institutional requirements. The patients/participants provided their written informed consent to participate in this study.

## Author contributions

Y-HS and J-YY derived the experimental results using the data. Y-HS proceeded to interpret the results of the experiment and wrote the manuscript. J-KS contributed to the interpretation and progress of the study as the corresponding author. YN, HJ, SS, and DN contributed to data curation. All authors contributed to the article and approved the submitted version.
